# Mutperiod: Analysis of periodic mutation rates in nucleosomes

**DOI:** 10.1016/j.csbj.2021.07.025

**Published:** 2021-07-26

**Authors:** Benjamin Morledge-Hampton, John J. Wyrick

**Affiliations:** a School of Molecular Biosciences, Washington State University, Pullman, WA 99164, USA; b Center for Reproductive Biology, Washington State University, Pullman, WA 99164, USA

**Keywords:** Mutations, DNA damage, DNA repair, Mismatch repair, Nucleosomes, Esophageal cancer, Software

## Abstract

Nucleosomes modulate DNA damage and repair, resulting in periodic mutation rates in nucleosomal DNA. Previous research has characterized these patterns in many sequenced tumor genomes; however, computational tools to identify and quantify these periodicities have not been developed for the broader scientific community. Here, we describe mutperiod, a Python and R based toolset that quantifies nucleosome mutational periodicities and compares them across different genetic and cellular backgrounds. We use mutperiod to demonstrate that DNA mismatch repair contributes to the nucleosome mutational periodicity observed in esophageal adenocarcinomas, and that the strength of this mutational periodicity varies in different chromatin states.

## Graphical abstract

**Figure:**
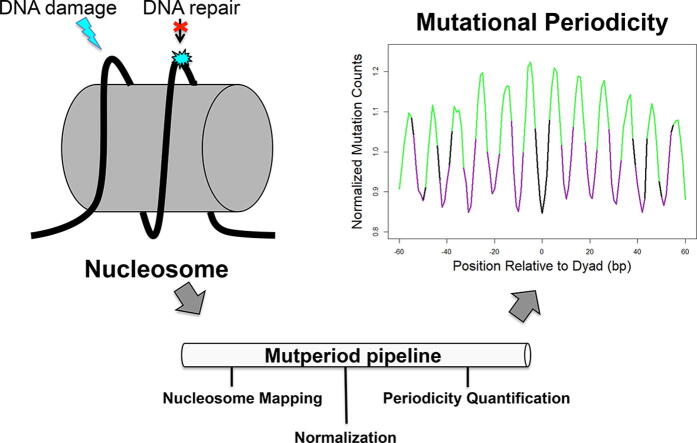

## 1 Introduction

Nucleosomes significantly impact DNA damage and repair, leading to two distinct periodic effects on mutation rates [1], [2]. First, DNA repair activity is inhibited within nucleosomes relative to linker DNA [1], often resulting in higher mutation rates at positions closer to the central dyad axis of nucleosomes. This mechanism creates a visible wave-like periodicity corresponding to the nucleosome repeat length (190 base pairs (bp) in human cells), resulting in what is termed translational periodicity [3]. Second, both DNA damage and repair can be modulated by the rotational setting of nucleosomal DNA. At positions where the minor groove of DNA faces the histone octamer, repair is generally inhibited, increasing mutation rates [2], [4]. In contrast, at positions where the minor groove faces away from the histone octamer, damage formation associated with ultraviolet (UV) light is increased, leading to the opposite mutation pattern in skin cancers such as melanoma [1], [2]. In either case, both mechanisms cause a periodic effect on mutation rates, called rotational periodicity, with a period corresponding to the 10.2 bp DNA helical repeat observed within nucleosomes [5].

Previous studies have characterized somatic mutation rates in nucleosomes for a variety of cancers and observed a wide range of both translational and rotational periodic effects [1], [2]. In some cases, these studies have used these periodicities to elucidate the molecular mechanism responsible for the observed mutational patterns. For example, analyses of these periodicities have revealed that differences in UV damage formation are responsible for elevated mutation rates at outward rotational settings in skin cancers [1], [2], while inhibition of base excision repair is likely responsible for elevated mutation rates at inward rotational settings in a variety of cancers [2], [4]. Hence, analyzing nucleosome mutational periodicities can provide key insights into the etiology of somatic mutations in different cancers.

However, software tools for quantifying nucleosome periodicities are limited and have previously not been developed with accessibility to the broader scientific community in mind. To meet this need, we developed a freely available software package called mutperiod. Mutperiod allows users to quantify, compare, and visualize nucleosome periodicities across distinct mutation data sets and is implemented with a robust user interface for maximal accessibility. We expect that the availability and flexibility of the mutperiod toolset will allow more researchers to analyze nucleosome periodicities in mutation data sets such as sequenced tumor genomes and better understand the interplay between chromatin and mutagenesis. In order to showcase these capabilities, we use mutperiod to show that mismatch repair contributes to nucleosome periodicity of somatic mutations in esophageal adenocarcinoma.

## 2 Experimental procedures

### 2.1 Availability and Installation of mutperiod

Source code and instructions for installation and use are available through the git repository at https://github.com/bmorledge-hampton19/mutperiod. In brief, mutperiod can be installed on Linux systems through the apt install command and the personal packaging archive at https://launchpad.net/~ben-morledge-hampton/+archive/ubuntu/mutperiod.

### 2.2 Input data required to run mutperiod

Mutperiod requires a genome fasta file, a nucleosome map, and mutation data as inputs in order to run. Specific information on the required formats for these inputs is present at the mutperiod git repository, linked above. The results in this paper were generated using mutation data from the International Cancer Genome Consortium [6], the hg19 genome assembly, and a nucleosome map of intergenic nucleosome positions called by Pich *et al*. from MNase-seq data [7]. These data are derived from lymphoblastoid cells, but have been used previously to map mutation data in a variety of cell types [2]. The code used to generate the nucleosome map is available at https://bitbucket.org/bbglab/nucleosome-periodicity/src/master/nucleosomes/. For the analysis of mutational periodicities in different chromatin domains, we used the entire set of called nucleosome positions (not just intergenic) derived from the MNase-seq data. The nucleosome positions were stratified using chromatin domains defined for either normal human lung fibroblasts (NHLF) or the GM12878 B-lymphocyte cell line [8].

### 2.3 Defining rotational and translational periodicities

For rotational periodicities, mutations are counted in a radius of 73 bp around the dyad center, corresponding to the 147 base pairs of DNA within the nucleosome [5]. For translational periodicities, the radius is set to 1000 to encompass multiple nucleosomes.

### 2.4 Quantification of periodicity

Mutperiod utilizes the Lomb-Scargle periodogram R package, lomb, to quantify the nucleosome mutational periodicities similarly to previous work [1], [2]. In order to produce meaningful periodograms, mutperiod requires that any input data have at least 5000 mutations within mapped nucleosome dyads. Periods between 5 bp and 25 bp are tested for rotational periodicity data and periods between 50 bp and 250 bp are tested for translational periodicity data. An oversampling factor of 100 is used. Mutperiod calculates a signal to noise ratio (SNR) representing the strength of the observed periodicity. The SNR is computed by dividing the period with maximum power (the signal) by the median power of all other periods not within 0.5 units of the maximum power period (the noise), as previously described [2].

### 2.5 Comparison of periodicities

Mutperiod facilitates the comparison of periodicities across different backgrounds or experimental conditions by first allowing the user to stratify mutation data cohorts at the beginning of the pipeline. Later in the pipeline, the separate cohorts are compared using the SNR values of the relevant periodicities. Either a permutation test (for aggregate data) or a Wilcoxon rank sum test (for individual tumor data) is used to determine the statistical significance of the comparison.

## 3 Results

### 3.1 Mutperiod analysis pipeline

Mutperiod processes mutation data through a hybrid Python and R pipeline. Users are expected to provide a genome fasta file, a nucleosome map, and mutation data as inputs. Mutperiod has native support for data from the ICGC portal or a slightly modified bed format that contains information on the nature of the mutations. These formats allow users to easily leverage available mutation data sets or adapt their own data for the pipeline. Mutation cohorts can be stratified out of the input data by various background conditions for comparison later in the pipeline. Mutperiod directly supports stratification of mutation data by microsatellite stability and mutation signature using previously developed R packages [9], [10]. Further stratifications can be manually designated by users in the mutation data input file.

Mutperiod uses the given inputs to count the mutations in and around nucleosomes, producing a table of mutation counts for each nucleosome position relative to the dyad center. Mutations can be counted within the radius of a single nucleosome for rotational periodicity or within several adjacent nucleosomes for translational periodicity. The resulting counts can be normalized in mutperiod using either the surrounding sequence context or a given mutation background from another input data set. After mutations are counted and optionally normalized, the relevant periodicities are quantified using a Lomb-Scargle periodogram, for which a signal-to-noise ratio is calculated [2].

**Fig. 1:**
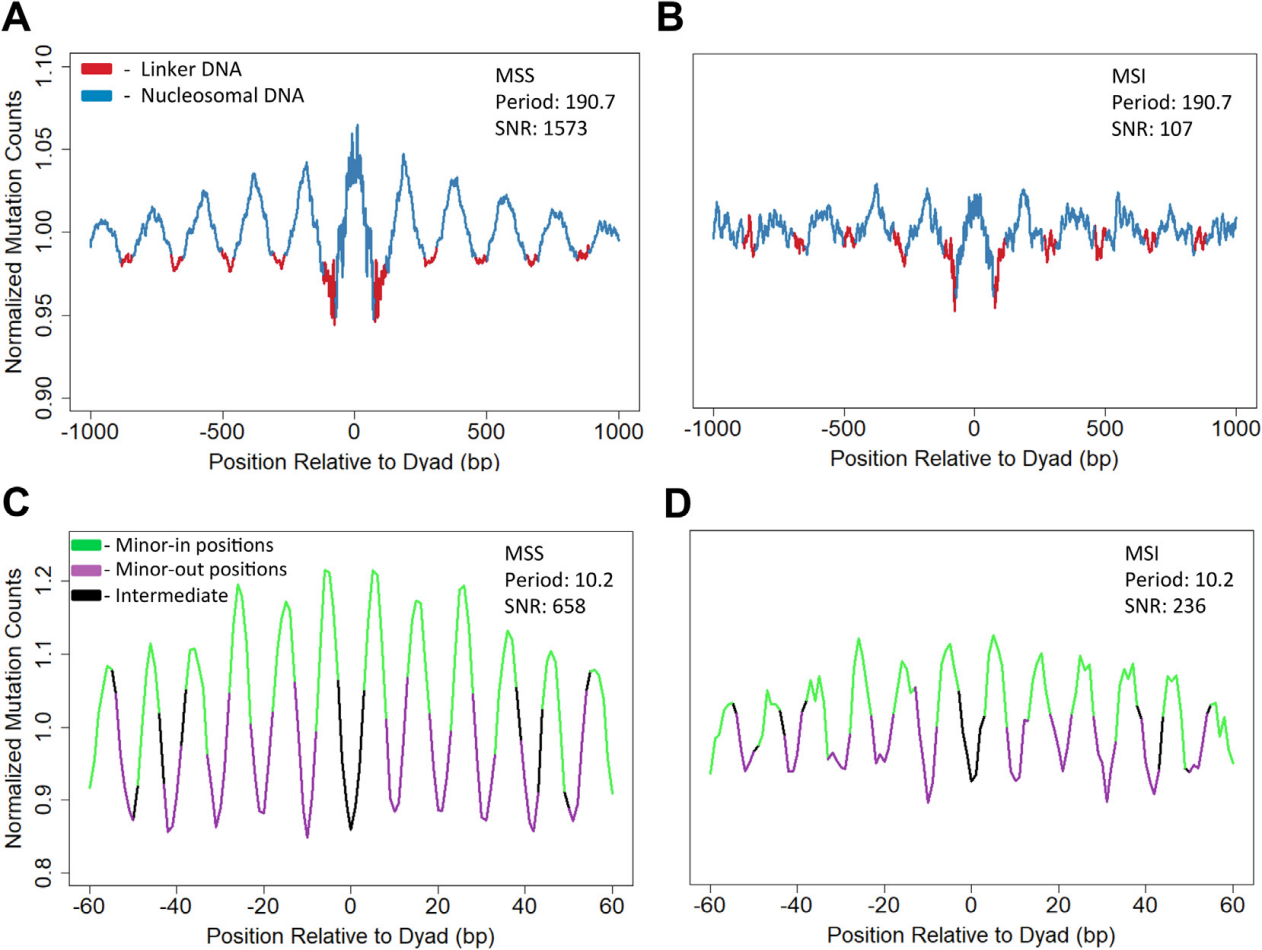
Microsatellite stability in esophageal tumors correlates with more pronounced nucleosome mutational periodicities. The translational (A, B) and rotational (C, D) mutational periodicities for esophageal tumor mutation data. All values are normalized by trinucleotide sequence context. Plots A and C represent microsatellite stable (MSS) tumors while plots B and D represent microsatellite instable (MSI) tumors. Translational data (A, B) is smoothed by averaging values in a sliding 11 bp window. Signal to noise ratio (SNR) and Period values were computed prior to smoothing. Non-smoothed data is available in Supplementary Fig. S5.

In order to investigate the link between nucleosomes and DNA damage and repair, it is vital to be able to compare the relative strengths of mutational periodicities across different genetic backgrounds, tumor types, or experimental conditions. Mutperiod can compare the strength of the periodicities between different data sets or in stratified subsets of a single data set (e.g., microsatellite stable versus instable tumors; see below) and determine if they are significantly different by either computing the SNR of user-generated random permutations of the aggregate mutation data or by using a Wilcoxon rank sum test for individual tumors. Furthermore, mutperiod can output plots of mutation counts with respect to the relevant periodicities to help users visualize them beyond the simple statistical output. These plots are similar to those presented in Fig. 1.

The entirety of the analysis pipeline supports an intuitive user interface through Python based Tkinter dialogs. In addition, a command line interface that installs with mutperiod can be used to manually pass inputs to the pipeline or simply invoke the user interface. Besides offering increased user accessibility, the user interface helps facilitate larger volumes of data processing by allowing the user to select multiple files or one or more directories to recursively search for all relevant files. All the selected files are then run through the pipeline in tandem.

### 3.2 Analysis of mutational periodicities in esophageal adenocarcinoma reveals a role for mismatch repair

We tested mutperiod’s ability to quantify differences in nucleosome mutational periodicities by analyzing the potential role of DNA mismatch repair in promoting these periodicities in esophageal adenocarcinomas (ESAD) [11]. Somatic mutations in ESAD display clear translational and rotational periodicities in nucleosomes [2], but the molecular mechanism responsible for these periodicities is unclear. In esophageal tumors, the translational periodicity is characterized by greater mutation rates in nucleosomes relative to linker DNA, and the rotational periodicity is characterized by greater mutation rates at positions where the minor groove of nucleosomal DNA faces inward, towards the histone octamer [2]. These periodicities suggest that repair inhibition may be an important mechanism [1], [2], [4]. The principal mutation signature associated with esophageal cancers is signature 17, which is characterized by an abundance of T > G mutations, as well as a lower frequency of C > T, T > A, and T > C mutations in certain sequence contexts [12]. Because T > G mutations are often linked to misincorporation of oxidized guanine opposite adenine during replication [13], we hypothesized that differential mismatch repair (MMR) in nucleosomes may play a role in creating the observed mutational periodicities.

To test this hypothesis, we used mutperiod to compare the periodicities of microsatellite stable (MSS) and instable (MSI) esophageal tumors. Microsatellite stability is an indicator of MMR proficiency [14], [15], as loss of MMR prevents the repair of replication errors in repetitive microsatellite regions, where they accumulate rapidly and lead to genomic instability. The data produced by mutperiod reveal markedly different periodicities across MSS and MSI tumors (Fig. 1). The SNR value for the aggregate MSS tumor data shows a nearly 15-fold increase compared to the MSI data for the translational periodicity (1573 vs. 107) and an almost 3-fold increase for the rotational periodicity (658 vs. 236).

The MSS data set contains approximately three times as many mutations mapped to nucleosomes as the MSI data set, which could affect the calculated SNR, since SNR tends to scale with sample size. To account for differences in sample size, we used mutperiod to analyze a subset of the aggregated MSS mutation data that matched the count of mutations (N = 2,921,950) in the MSI data set. For both translational and rotational periodicities, the subset of the MSS mutation data still had much higher SNR than the MSI data (Supplementary Fig. S1). We repeated this analysis for 100 random subsets of the MSS data. In all cases, the SNR of the MSS subset was much higher than the MSI data (Supplementary Fig. S2).

**Fig. 2:**
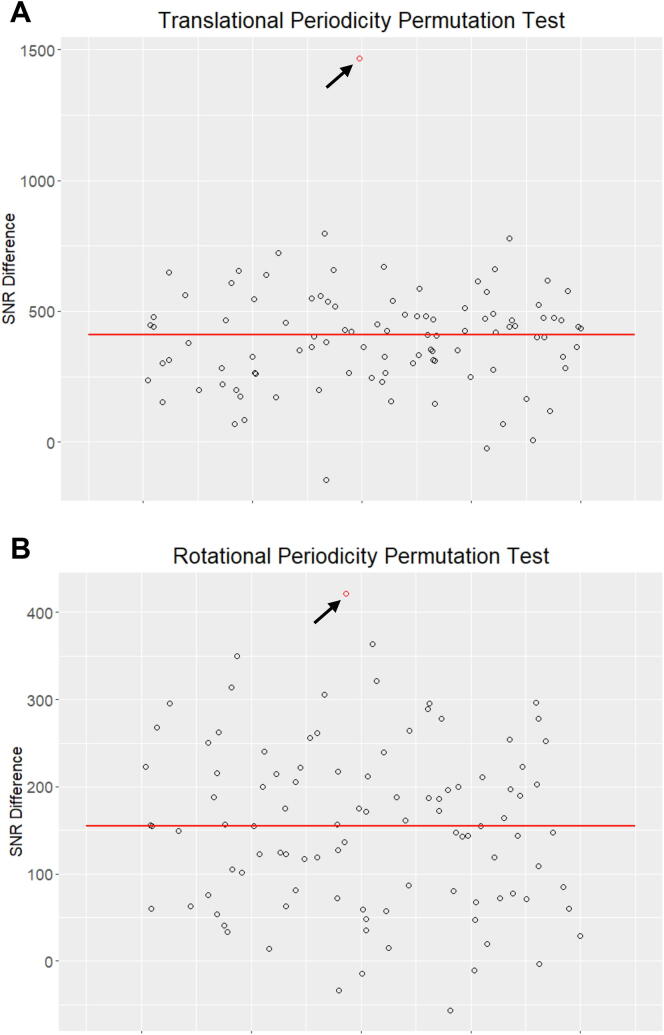
Permutation analysis of the differences in SNR for aggregate mutation data. The distribution of the difference in signal to noise ratio (SNR) values for 100 random permutations of the aggregate MSS and MSI data. Permutations preserve the difference in the number of mutations between the MSS and MSI data. Plots show the distribution of SNR differences for (A) translational periodicity and (B) rotational periodicity. Red bars represent the median of each data set and the red points highlighted by the arrows represents the difference between the original MSS vs MSI data (Fig. 1). (For interpretation of the references to colour in this figure legend, the reader is referred to the web version of this article.)

To confirm that the differences in SNR between the MSS and MSI mutation data were statistically significant, we performed a permutation test. We randomly permutated the MSS and MSI labels for the aggregated mutation data, while maintaining the same number of mutations in the permuted MSS and MSI classes, and calculated the difference in SNR using mutperiod. It is interesting to note that the median difference in SNR in the permuted classes was greater than zero (Fig. 2), likely reflecting the higher SNR in the permuted class with more mutations (e.g., the permuted MSS class). Across 100 random permutations of the aggregate data, all produced a lower difference in SNR than observed between MSS and MSI tumors for both rotational and translational periodicities (*P* < 0.01 for both cases; Fig. 2).

**Fig. 3:**
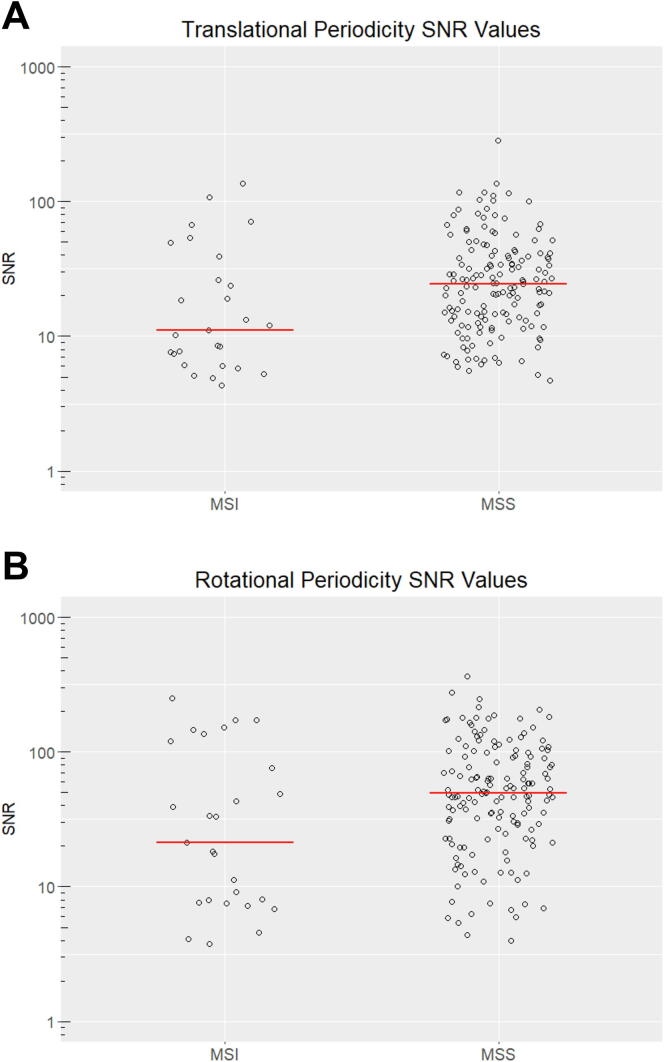
Microsatellite stable tumors have more pronounced nucleosome periodicities. The distribution of signal to noise (SNR) values for individual esophageal tumor mutation data sets categorized as either microsatellite instable (MSI, n = 27) or microsatellite stable (MSS, n = 153). Plots show the distribution of (A) translational periodicity and (B) rotational periodicity SNR values. Red bars represent the median of each data set. (For interpretation of the references to colour in this figure legend, the reader is referred to the web version of this article.)

In addition to the analysis of the aggregate data, we analyzed the distributions of SNR values for individual MSS and MSI tumors. The data indicated that individual MSS tumors had a significantly higher translational (*P* = 0.010) and rotational (*P* = 0.030) periodicity than MSI tumors (Fig. 3), even though MSI tumors on average had a greater number of mutations. In summary, these results indicate that mismatch repair status is significantly associated with the strength of mutational periodicity in nucleosomes, both in the aggregate mutation data and in individual tumors.

**Fig. 4:**
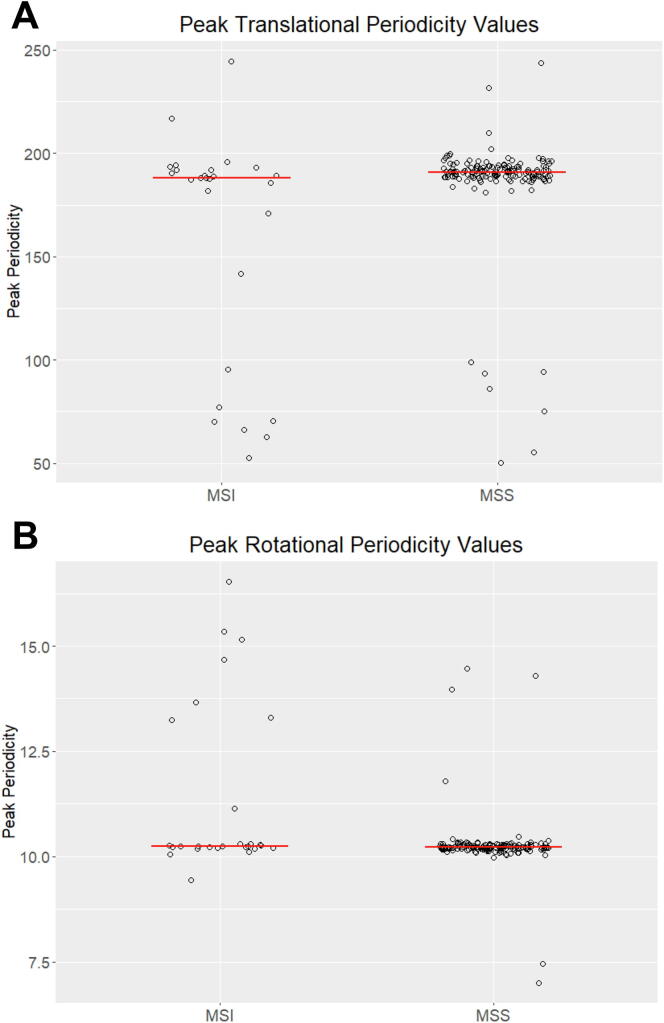
Microsatellite instable tumors more frequently deviate from the expected peak periodicity value. The distribution of peak periodicity values for individual esophageal tumor mutation data sets categorized as either microsatellite instable (MSI, n = 27) or microsatellite stable (MSS, n = 153). Peak periodicity is defined as the period with the highest power, as determined by a Lomb-Scargle periodogram. Only periods between 50 bp and 250 bp were analyzed for translational periodicity (A) and only periods between 5 bp and 25 bp were analyzed for rotational periodicity (B). Red bars represent the median of each data set. (For interpretation of the references to colour in this figure legend, the reader is referred to the web version of this article.)

When analyzing the period with the maximum power in each tumor, we found that while many tumors in the MSI data set have the expected median periods of ~190 bp and 10.2 bp in translational and rotational data, respectively, they also have a much greater variability in the peak periodicity values compared to the MSS tumors (Fig. 4). This is particularly apparent for translational periodicities, where the MSI tumors had a much higher standard deviation (SD = 56) and interquartile range (IQR = 74) in the values of the peak periodicity than the MSS tumors (SD = 24.83; IQR = 4.56). There was a similarly elevated variability of peak rotational periodicities for the MSI tumors (SD = 2.49) relative to the MSS tumors (SD = 0.68). This is consistent with previous results, indicating that MSI tumors may display a range of MMR-deficient phenotypes [16], which could account for the variability in periodicity values. The outliers among the MSI samples may represent tumors where MMR was disabled earlier in tumorigenesis or more severely. Taken together, these findings suggest that the nucleosomal mutational periodicities observed in ESAD tumors are associated with proficient mismatch repair.

### 3.3 Nucleosomal mutation periodicities vary in different chromatin domains

The analysis described above used a nucleosome map encompassing all intergenic regions of the human genome. However, other analyses may benefit from a more targeted nucleosome map. To address this need, mutperiod is equipped with the ability to stratify nucleosome maps using a bed file of genomic loci. Possible targets for stratification include genomic regions with distinct patterns of histone post-translational modifications or chromatin states. As an example, we stratified the complete nucleosome map (i.e., intergenic and genic nucleosomes) into nucleosome subsets associated with either heterochromatin or transcribed domains, as defined in a previous study [8].

**Fig. 5:**
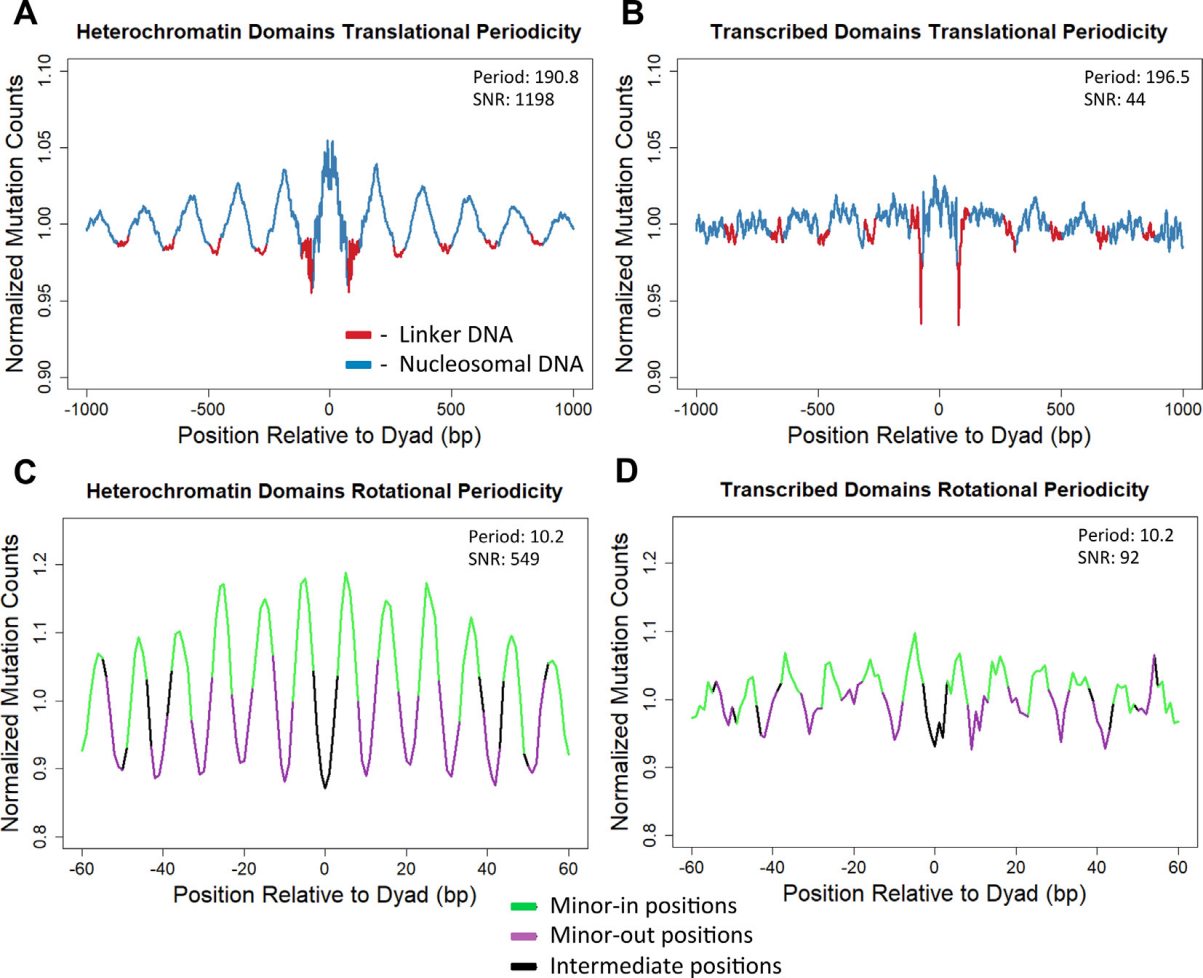
Analysis of nucleosome mutation periodicities in distinct chromatin domains. (A, B) The translational and (C, D) rotational mutational periodicities for esophageal tumor mutation data under different nucleosome map stratification conditions. All values are normalized by trinucleotide sequence context. Plots A and C were generated using a nucleosome map stratified by normal human lung fibroblast (NHLF) heterochromatin domains while plots B and D use a map stratified by NHLF transcribed domains. Translational data (A, B) is smoothed by averaging values in a sliding 11 bp window to reduce the visual clutter caused by rotational periodicity. SNR and Period values were computed prior to smoothing.

The differences between mutational periodicities in heterochromatin vs. transcribed regions were striking, with the SNR of mutational periodicities in transcribed regions being significantly less than for nucleosomes in heterochromatin domains (Fig. 5). Since there were more nucleosomes associated with heterochromatin domains, we analyzed a subset of heterochromatin domain nucleosomes that matched the count for transcribed regions (Supplementary Fig. S3). The translational and rotational SNR’s for the heterochromatin subset were still much higher than those of the transcribed domain nucleosomes (compare Fig. 5B,D with Supplementary Fig. S3). A similar stratification using a chromatin map from a different cell type (i.e., GM12878 B-lymphocyte cell line instead of normal human lung fibroblasts) showed similar results (Supplementary Fig. S4). In summary, mutperiod can be used to characterize mutation periodicities in distinct genomic or chromatin domains. In this case, our analysis suggests that in esophageal adenocarcinomas, nucleosome mutation periodicities are more prominent in heterochromatin, and less prominent in transcribed genes.

## 4 Discussion

While recent studies have highlighted the prevalence and importance of periodic mutation rates in nucleosomes, accessible software tools to analyze these mutational periodicities have been lacking. To meet this need, we have developed an accessible and freely available software package called mutperiod. We use mutperiod to show that DNA mismatch repair (MMR) contributes to periodic mutation rates in esophageal adenocarcinomas, highlighting the power of mutperiod in identifying new mechanisms that contribute to mutational periodicity in nucleosomes.

A key feature of mutperiod is its ease of use and accessibility. It is well known that easily accessible software tools are more likely to be used than inaccessible tools by members of the scientific community [17], [18]. We have taken special care to design mutperiod with ease of access in mind, in order to encourage other researchers to leverage nucleosome periodicities in their work and to increase the efficiency of their research. Much of this accessibility comes from a simple installation protocol and an intuitive user interface. Moreover, mutperiod is lightweight, and the above analysis of esophageal tumor data was run in a single day without the use of a computing cluster or any particularly expensive hardware (omitting the permutation test, which was much more computationally expensive). For this analysis, much of the runtime comes from the external package, MSISeq [9], which is beyond the scope of this research to optimize. The next most computationally expensive portion of the pipeline comes from mapping individual mutations to the given nucleosome map. This process has been optimized by sorting both inputs and comparing across each in parallel, guaranteeing worst-case linear runtime. We are confident that mutperiod can be leveraged even by researchers with limited computational experience and resources.

Somatic mutations in esophageal adenocarcinomas have among the strongest rotational and translational periodicities in nucleosomes of any cancer type [2], but the molecular mechanism(s) responsible for these mutational periodicities have been unclear. It has been previously suggested that differential base excision repair (BER) may be responsible for these mutational periodicities [2], since BER is regulated by both the translational and rotational positioning of the DNA base lesion in the nucleosome [4], [19], and because many oxidative DNA lesions, which are associated with mutagenesis in esophageal tumors [20], [21], are repaired by BER [22]. However, the principal mutation signature associated with esophageal cancers (signature 17) has been linked to misincorporation of 8-oxoguanine (8-oxoG) nucleotides opposite adenine during replication [13]. BER enzymes such as hOGG1 are unable to efficiently repair 8-oxoG lesions paired with adenine [23]; instead, these misincorporated 8-oxoG lesions are likely repaired by MMR [24], [25]. Our results support the hypothesis that inhibition of MMR in nucleosomes contributes to mutational periodicities in esophageal tumors. This hypothesis is supported by a recent report that colorectal cancers with DNA polymerase epsilon mutator alleles, which generates DNA replication errors that are repaired by MMR, also show a rotational mutation periodicity in nucleosomes [26]. Our results are consistent with biochemical studies demonstrating that nucleosomes inhibit mismatch repair [27] and showcase the power of mutperiod in elucidating this relationship in human tumors.

Our analysis using mutperiod also demonstrates that the observed nucleosome mutation patterns are much more prominent in heterochromatin than in transcribed genes. This suggests that these mutation periodicities may not significantly impact the occurrence of potential driver mutations in esophageal adenocarcinoma, since these driver mutations typically occur in transcribed exons. A key question is what molecular mechanism causes this difference mutational periodicity in heterochromatin versus transcribed euchromatin. One possibility is that nucleosome positioning is more dynamic in transcribed domains, either within individual cells or between different cell types, resulting in weaker nucleosome periodicities. Alternatively, it is possible that transcription-coupled repair of oxidative damage [28] may diminish the impact of nucleosomes on mutation rates in transcribed domains. Moreover, transcribed DNA is known to be enriched for histone H3 K36 methylation, which can recruit mismatch repair proteins and influence mutagenesis [29], [30]. It is possible that high levels of H3 K36 methylation promote efficient mismatch repair in transcribed nucleosomes, thereby diminishing the impact of nucleosomes on mutagenesis. While future studies will be needed to test these hypotheses, our findings highlight the utility of mutperiod in characterizing mutation patterns in nucleosomal DNA.

## CRediT authorship contribution statement

**Benjamin Morledge-Hampton:** Conceptualization, Data curation, Formal analysis, Investigation, Methodology, Software, Validation, Writing - original draft. **John J. Wyrick:** Conceptualization, Funding acquisition, Project administration, Writing - review & editing.

## Declaration of Competing Interest

The authors declare that they have no known competing financial interests or personal relationships that could have appeared to influence the work reported in this paper.

## References

[1] Brown AJ, Mao P, Smerdon MJ, Wyrick JJ, Roberts SA. Nucleosome positions establish an extended mutation signature in melanoma. PLOS Genet. 2018;14: e1007823. Available: 10.1371/journal.pgen.1007823. 10.1371/journal.pgen.1007823 PMC6287878 30485262

[2] Pich O., Muiños F., Sabarinathan R., Reyes-Salazar I., Gonzalez-Perez A., Lopez-Bigas N. (2018). Somatic and germline mutation periodicity follow the orientation of the DNA minor groove around nucleosomes. Cell.

[3] Valouev A., Johnson S.M., Boyd S.D., Smith C.L., Fire A.Z., Sidow A. (2011). Determinants of nucleosome organization in primary human cells. Nature.

[4] Mao P., Brown A.J., Malc E.P., Mieczkowski P.A., Smerdon M.J., Roberts S.A. (2017). Genome-wide maps of alkylation damage, repair, and mutagenesis in yeast reveal mechanisms of mutational heterogeneity. Genome Res.

[5] Luger K., Mäder A.W., Richmond R.K., Sargent D.F., Richmond T.J. (1997). Crystal structure of the nucleosome core particle at 2.8 Å resolution. Nature.

[6] Zhang J., Bajari R., Andric D., Gerthoffert F., Lepsa A., Nahal-Bose H. (2019). The international cancer genome consortium data portal. Nat Biotechnol.

[7] Gaffney D.J., McVicker G., Pai A.A., Fondufe-Mittendorf Y.N., Lewellen N., Michelini K. (2012). Controls of nucleosome positioning in the human genome. PLoS Genet.

[8] Ernst J., Kheradpour P., Mikkelsen T.S., Shoresh N., Ward L.D., Epstein C.B. (2011). Mapping and analysis of chromatin state dynamics in nine human cell types. Nature.

[9] Ni Huang M., McPherson J.R., Cutcutache I., Teh B.T., Tan P., Rozen S.G. (2015). MSIseq: software for assessing microsatellite instability from catalogs of somatic mutations. Sci Rep.

[10] Rosenthal R., McGranahan N., Herrero J., Taylor B.S., Swanton C. (2016). deconstructSigs: delineating mutational processes in single tumors distinguishes DNA repair deficiencies and patterns of carcinoma evolution. Genome Biol.

[11] Consortium IP-CA of WG (2020/02/05.). Pan-cancer analysis of whole genomes. Nature.

[12] Alexandrov L.B., Nik-Zainal S., Wedge D.C., Aparicio S.A.J.R., Behjati S., Biankin A.V. (2013). Signatures of mutational processes in human cancer. Nature.

[13] Tomkova M., Tomek J., Kriaucionis S., Schuster-Böckler B. (2018). Mutational signature distribution varies with DNA replication timing and strand asymmetry. Genome Biol.

[14] Boyer JC, Umar A, Risinger JI, Lipford JR, Kane M, Yin S, et al. Microsatellite Instability, Mismatch Repair Deficiency, and Genetic Defects in Human Cancer Cell Lines. Cancer Res. 1995;55: 6063 LP – 6070. Available: http://cancerres.aacrjournals.org/content/55/24/6063.abstract. 8521394

[15] Honecker F., Wermann H., Mayer F., Gillis A.J.M., Stoop H., van Gurp R.J.L.M. (2009). Microsatellite instability, mismatch repair deficiency, and BRAF mutation in treatment-resistant germ cell tumors. J Clin Oncol..

[16] Frigola J., Sabarinathan R., Mularoni L., Muiños F., Gonzalez-Perez A., López-Bigas N. (2017/11/06.). Reduced mutation rate in exons due to differential mismatch repair. Nat Genet.

[17] Macaulay C., Sloan D., Jiang X., Forbes P., Loynton S., Swedlow J.R. (2009). Usability and user-centered design in scientific software development. IEEE Softw.

[18] Shachak A., Shuval K., Fine S. (2007). Barriers and enablers to the acceptance of bioinformatics tools: a qualitative study. J Med Libr Assoc.

[19] Rodriguez Y., Smerdon M.J. (2013). The structural location of DNA lesions in nucleosome core particles determines accessibility by base excision repair enzymes. J Biol Chem.

[20] Lee J.S., Oh T.Y., Ahn B.O., Cho H., Kim W.B., Kim Y.B. (2001). Involvement of oxidative stress in experimentally induced reflux esophagitis and Barrett’s esophagus: clue for the chemoprevention of esophageal carcinoma by antioxidants. Mutat Res Mol Mech Mutagen.

[21] Chen X., Ding Y.W., Yang G., Bondoc F., Lee M.-J., Yang C.S. (2000). Oxidative damage in an esophageal adenocarcinoma model with rats. Carcinogenesis.

[22] Friedberg EC, Walker GC, Siede W, Wood RD, Schultz RA, Ellenberger T. DNA Repair and Mutagenesis. 2nd ed. ASM Press; 2006. doi:10.1128/9781555816704

[23] Bjørås M., Luna L., Johnsen B., Hoff E., Haug T., Rognes T. (1997). Opposite base-dependent reactions of a human base excision repair enzyme on DNA containing 7,8-dihydro-8-oxoguanine and abasic sites. EMBO J..

[24] Colussi C., Parlanti E., Degan P., Aquilina G., Barnes D., Macpherson P. (2002). The mammalian mismatch repair pathway removes DNA 8-oxodGMP incorporated from the oxidized dNTP pool. Curr Biol..

[25] Egashira A., Yamauchi K., Yoshiyama K., Kawate H., Katsuki M., Sekiguchi M. (2002). Mutational specificity of mice defective in the MTH1 and/or the MSH2 genes. DNA Repair (Amst)..

[26] Fang H, Barbour JA, Poulos RC, Katainen R, Aaltonen LA, Wong JWH. Mutational processes of distinct POLE exonuclease domain mutants drive an enrichment of a specific TP53 mutation in colorectal cancer. PLOS Genet. 2020;16: e1008572. Available: 10.1371/journal.pgen.1008572 10.1371/journal.pgen.1008572 PMC7018097 32012149

[27] Li F., Tian L., Gu L., Li G.-M. (2009). Evidence that nucleosomes inhibit mismatch repair in eukaryotic cells. J Biol Chem..

[28] Banerjee D., Mandal S.M., Das A., Hegde M.L., Das S., Bhakat K.K. (2011). Preferential repair of oxidized base damage in the transcribed genes of mammalian cells. J Biol Chem..

[29] Gonzalez-Perez A., Sabarinathan R., Lopez-Bigas N. (2019). Local determinants of the mutational landscape of the human genome. Cell.

[30] Huang Y., Li G.-M. (2020). DNA mismatch repair in the chromatin context: Mechanisms and therapeutic potential. DNA Repair (Amst)..

