# Supplementary material for: Mutperiod: Analysis of periodic mutation rates in nucleosomes
Source: Comput Struct Biotechnol J. 2021 Jul 26;19:4177–83. doi: 10.1016/j.csbj.2021.07.025 (PMC8349767; doi:10.1016/j.csbj.2021.07.025)
Supplement: Supplementary data 1 [file mmc1.pdf]

Supplemental Materials

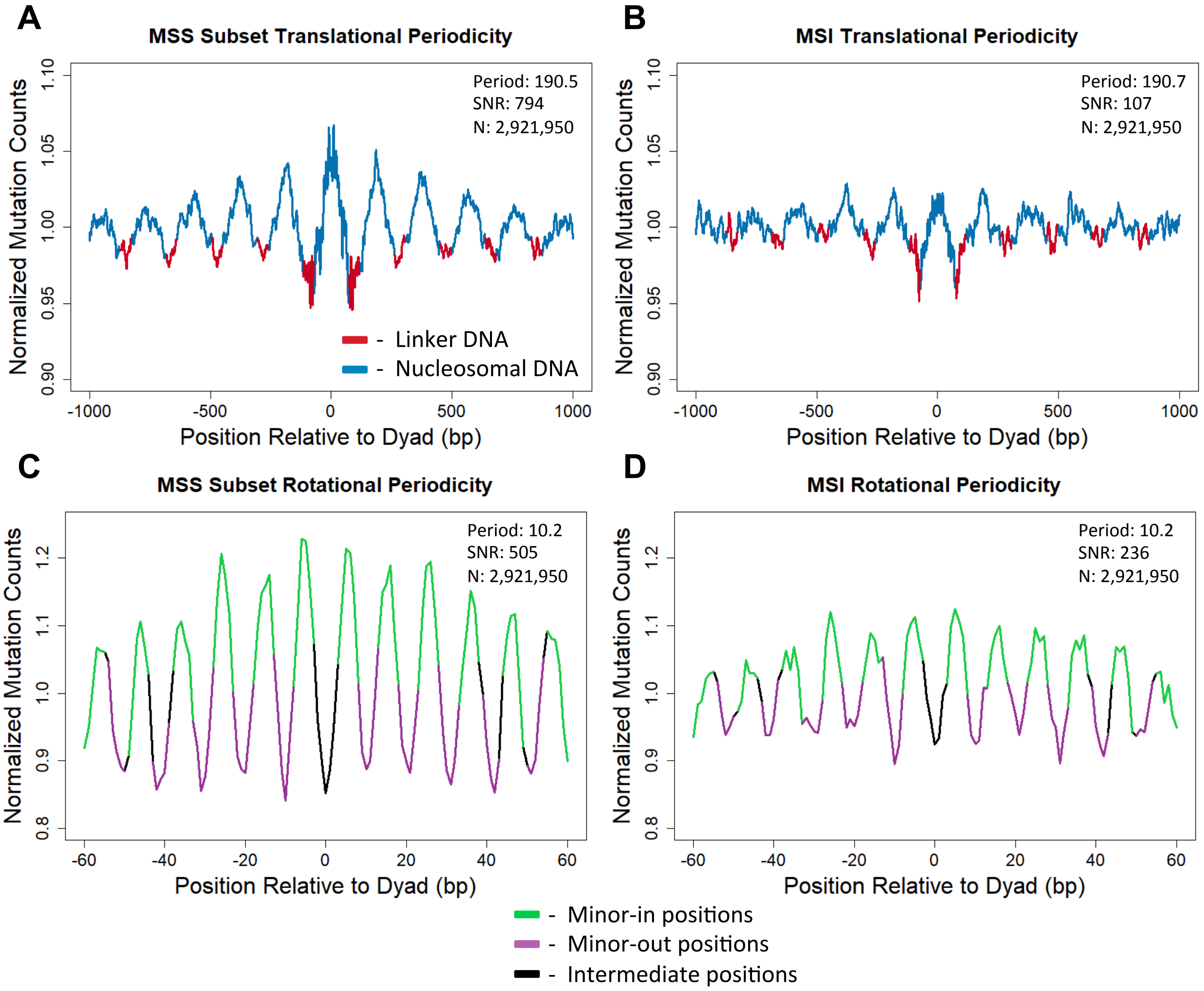

Supplementary Figure S1: Differences in mutation counts between microsatellite stable and instable tumors do not account for differences in periodicities. The translational (**A**, **B**) and rotational (**C**, **D**) mutational periodicities for esophageal tumor mutation data. All values are normalized by trinucleotide sequence context. Plots **A** and **C** represent microsatellite stable (MSS) tumors while plots **B** and **D** represent microsatellite instable (MSI) tumors. Mutations from MSS tumors were randomly sampled to be equal in number to those in MSI tumors. Translational data (**A**, **B**) is smoothed by averaging values in a sliding 11 bp window. SNR and Period values were computed prior to smoothing.

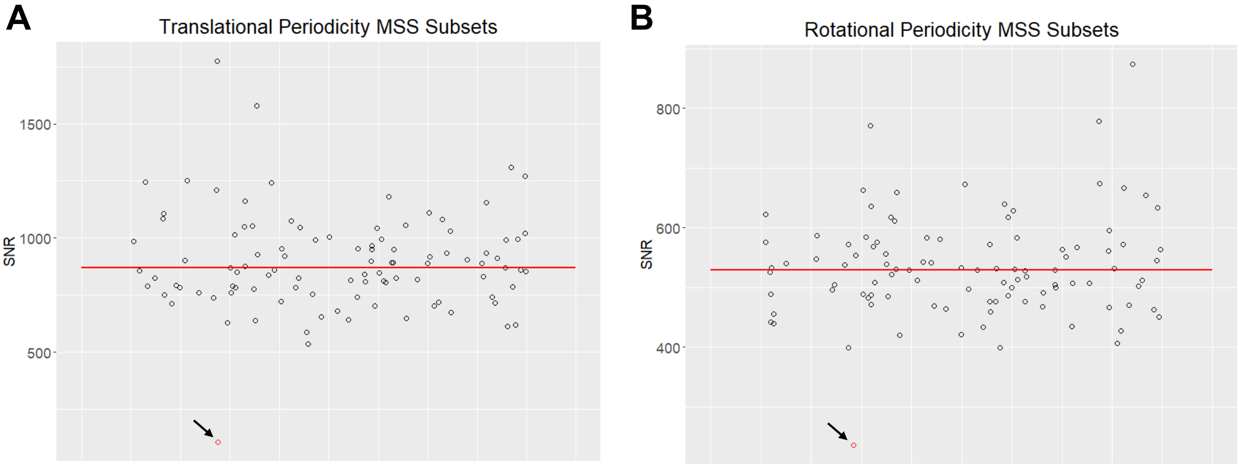

Supplementary Figure S2: Distribution of SNRs for MSS Subsets. Random subsets of the MSS data equal in size to the MSI data were taken and signal to noise ratios were calculated for translational (**A**) and rotational (**B**) periodicities. Red bars represent the median of each data set and the red points highlighted by the arrows represent the SNR for the MSI data.

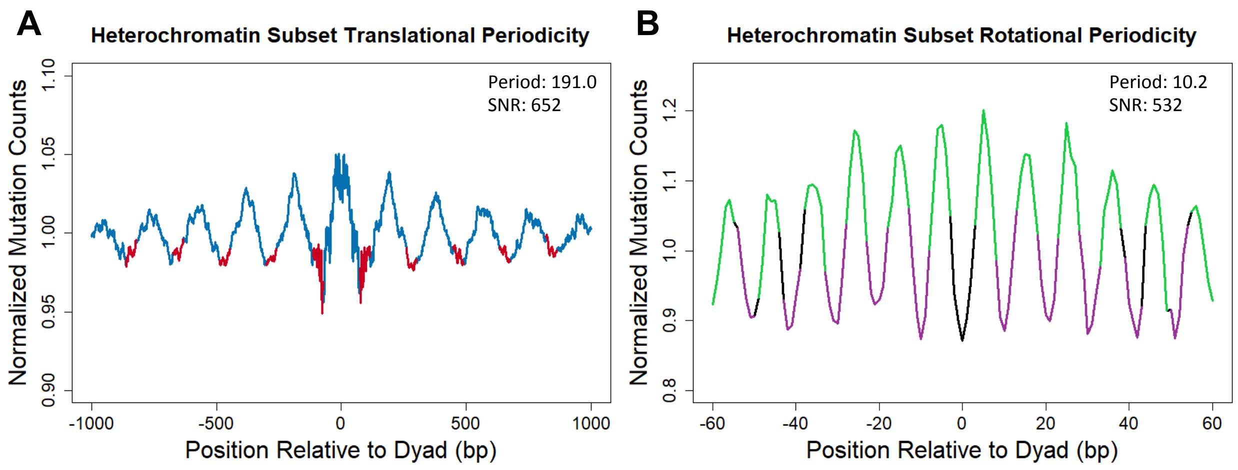

Supplementary Figure S3: Subset of nucleosome map stratified by NHLF chromatin domains. The translational (**A**) and rotational (**B**) mutational periodicities for esophageal tumor mutation data under a nucleosome map which is a subset of NHLF heterochromatin nucleosomes equal in size to the transcription nucleosome map (n = 2,277,747). This data was generated in order to validate that the observed differences are not purely due to differences in the sizes between each map.

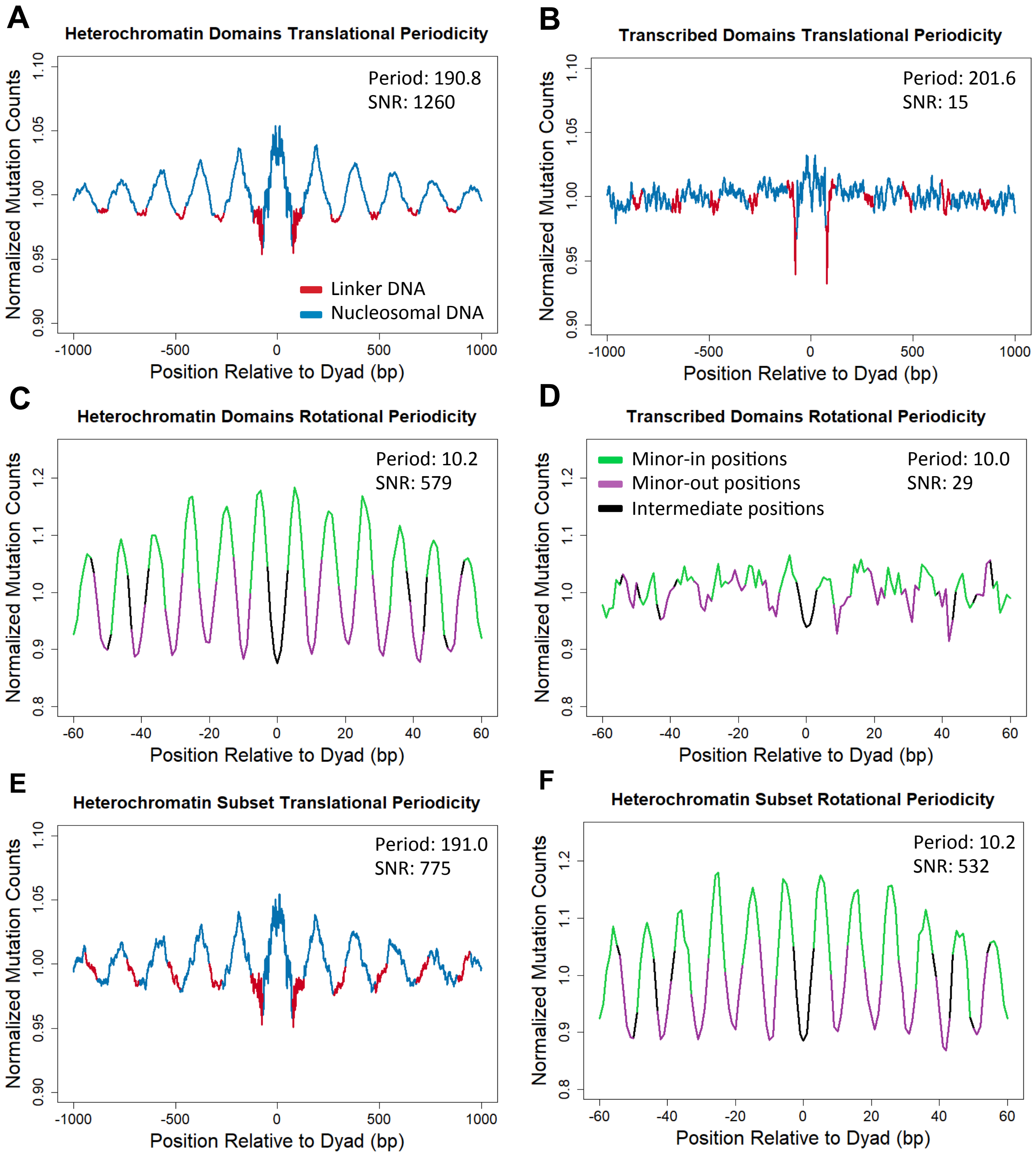

Supplementary Figure S4: Analysis of mutation periodicities in nucleosome map stratified by GM12878 chromatin domains. The translational (**A**, **B**, **E**) and rotational (**C**, **D**, **F**) mutational periodicities for esophageal tumor mutation data under different nucleosome map stratification conditions. All values are normalized by trinucleotide sequence context. Plots **A** and **C** were generated using a nucleosome map stratified by GM12878 heterochromatin domains while plots **B** and **D** use a map stratified by GM12878 transcribed domains. Plots **E** and **F** use a subset of heterochromatin nucleosomes equal in size to the transcription nucleosome map (n = 2,070,606) in order to validate that the observed differences are not purely due to differences in

the sizes between each map. Translational data (**A**, **B**, **E**) is smoothed by averaging values in a sliding 11 bp window to reduce the visual clutter caused by rotational periodicity. SNR and Period values were computed prior to smoothing.

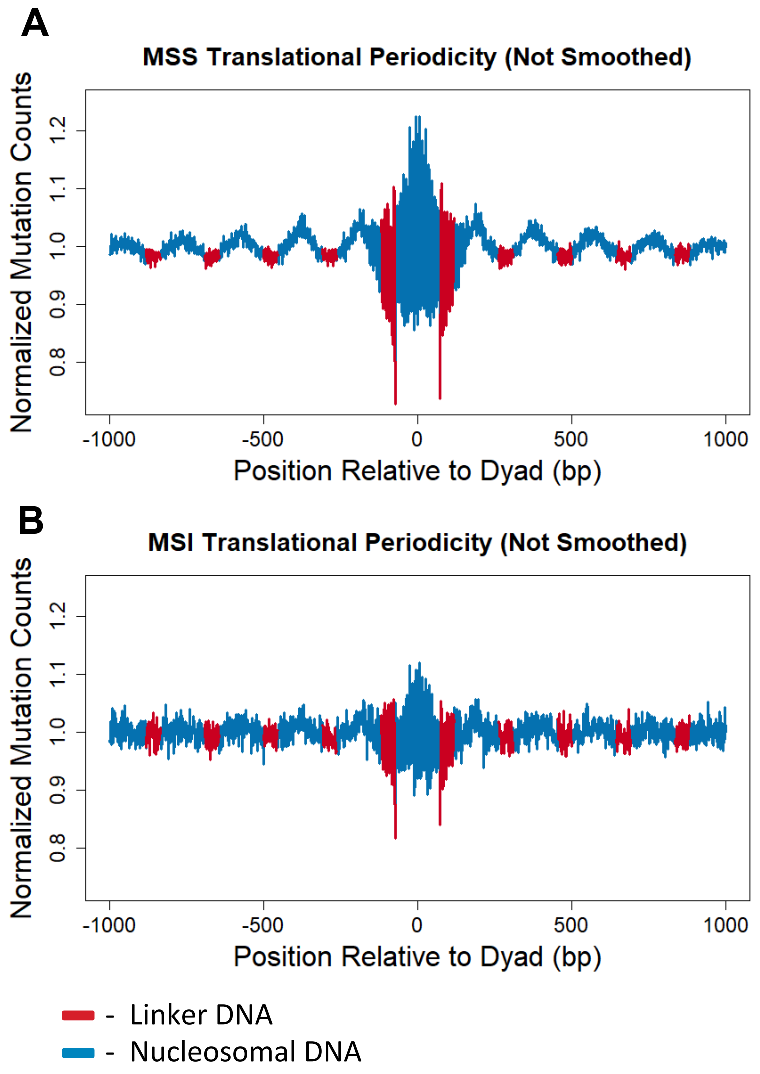

Supplementary Figure S5: Unsmoothed translational periodicity data. The translational periodicities for MSS (**A**) and MSI (**B**) esophageal tumor mutation data. All values are normalized by trinucleotide sequence context. The underlying data are identical to those in **Fig. 1A** and **1B**, except that no smoothing was applied prior to figure generation. Axes were expanded compared to **Fig. 1A** and **1B** in order to accommodate the larger range of normalized mutation counts.
